# Macrophages are recruited to hypoxic tumor areas and acquire a Pro-Angiogenic M2-Polarized phenotype via hypoxic cancer cell derived cytokines Oncostatin M and Eotaxin

**DOI:** 10.18632/oncotarget.2110

**Published:** 2014-06-17

**Authors:** Chakrapani Tripathi, Brij Nath Tewari, Ranjana Kumari Kanchan, Khemraj Singh Baghel, Naveen Nautiyal, Richa Shrivastava, Harbeer Kaur, Madan Lal Bramha Bhatt, Smrati Bhadauria

**Affiliations:** ^1^ Division of Toxicology, Central Drug Research Institute, (CSIR) Lucknow, India; ^2^ Academy of Scientific Innovative Research, (AcSIR) India; ^3^ Department of Radiotherapy, King George Medical University, Lucknow, India; ^4^ Department of Surgical Oncology, King George Medical University, Lucknow, India

**Keywords:** Hypoxia, M2-Polarization, TAM, Tumor-microenvironment, Chemoattract, Pro-angiogenic, Breast Cancer

## Abstract

TAMs, a unique and distinct M2-skewed myeloid population of tumor stroma, exhibiting pro-tumor functions is fast emerging as a potential target for anti-cancer immunotherapy. Macrophage-recruitment and M2-polarization represent key TAMs-related phenomenon that are amenable to therapeutic intervention. However successful translation of these approaches into effective therapeutic regimen requires better characterization of tumor-microenvironment derived signals that regulate macrophage recruitment and their polarization. Owing to hypoxic milieu being a persistent feature of tumor-microenvironment and a major contributor to malignancy and treatment resistance, the current study was planned with an aim to decipher tumor cell responses to hypoxia vis-a-vis macrophage homing and phenotype switching. Here, we show that hypoxia-primed cancer cells chemoattract and polarize macrophages to pro-angiogenic M2-polarized subtype via Eotaxin and Oncostatin M. Concordantly, hypoxic regions of human breast-cancer specimen exhibited elevated Eotaxin and Oncostatin M levels with concurrently elevated M2-macrophage content. Blockade of Eotaxin/Oncostatin M not only prevented hypoxic breast-cancer cells from recruiting and polarizing macrophages towards an M2-polarized phenotype and retarded tumor progression in 4T1/BALB/c-syngenic-mice-model of breast-cancer but also enhanced the efficacy of anti-angiogenic Bevacizumab. The findings established these two cytokines as novel targets for devising effective anticancer therapy particularly for tumors that are refractory or develop resistance to anti-angiogenic therapeutics.

## INTRODUCTION

The extremely complex tumor microenvironment is composed of not only an expanding population of transformed cells; it also includes stromal cells such as smooth muscle cells, fibroblasts and macrophages [1]. Together these stromal cells act as prominent modifiers of tumor growth and progression [2]. The propensity of tumors to progress does not solely emanate from oncogenic transformations occurring within cancer cells. It is also a cumulative manifestation of ongoing dynamic interactions between tumor cells and surrounding stromal cells [3]. Macrophages are one of the major populations of tumor infiltrating immune cells and represent the most abundant host cell population within tumor stroma [4, 1]. Cytotoxicity of macrophages during the early immune response contributes to tumor killing, however in most solid tumors, macrophages inversely affect prognosis by potentiating cancer progression [5]. The resident macrophages of tumor stroma exhibiting pro-tumor functions are often termed as tumor associated macrophages (TAMs). Clinical studies have revealed positive correlation between presence of TAMs and adverse clinical outcome/ shorter survival in various cancer types, including non-small cell lung cancer [6, 7], breast cancer [8] and Hodgkin's lymphoma. Studies employing experimental mice models revealed that TAMs facilitate tumor progression by promoting inflammation, stimulating tumor neoangiogenesis, enhancing tumor cell dissemination, potentiating metastasis and suppressing antitumor immunity [9-13]. TAMs are short lived and do not proliferate *insitu*, consequently, their repertoire must be continuously replenished throughout the cancer progression [14-16]. Macrophage infiltration is thus a critical requisite for TAMs assisted tumor progression and targeting this critical event might retard tumor progression. In agreement with this, hindering macrophage infiltration into tumors of colony-stimulating factor-1-knockout mice bearing the polyoma middle T oncoprotein markedly reduced the progression to malignancy [17]. Furthermore, counteracting the infiltration of TAM by antagonizing the key chemokine mediator of macrophage recruitment i.e. CCL5 markedly reduced tumor infiltrate and reduced tumor growth, thereby emphasizing the importance of recruitment of TAMs during tumor progression [18].

Macrophages exhibit tremendous degree of plasticity in their responses during the course of physiological processes and pathophysiological progression by undergoing a rapid phenotype switching [19]. The microenvironmental cues elicited during such progressions, activate macrophages to acquire functionally distinct phenotypes [20]. Two well-established polarized phenotypes are classically activated macrophages (M1-macrophages) and alternatively activated macrophages (M2 macrophages) [21, 22]. IFN-γ, TNF- α and microbial ligands such as LPS elicit a classically activated M1 form of macrophages. They possess strong antigen presenting property and affect microbicidal and tumoricidal response by (i) producing proinflammatory mediators such as nitric oxide, ROS and (ii) by inducing Th1 immunity through production of cytokine such as IL-12 and IL-23. In contrast, IL-4, IL-13, glucocorticoids, IL-10, IgG complexes and Toll-like receptor ligands elicit the alternatively activated M2 form of macrophages. The various subtypes of M2 macrophages have poor antigen presenting capability. They effectively suppress T cell activation and exhibit little or no cytotoxicity for tumor cells because of their limited potential to produce NO and proinflammatory cytokines. Rather they exhibit pro-tumor functions such as potentiating tumor neo-angiogenesis, metastasis and tissue remodeling. The TAMs express marker profile that is characteristic of alternatively activated M2 macrophages [1]. Although the macrophage infiltration and tumor dependent M2 polarization of the infiltrated macrophages are amongst the key phenomenon governing cancer progression and therapeutic efficacy, the origin of these responses are so far unclear. Gaining better insights into the origin and regulation of these responses will provide means to selectively target or reprogram TAMs so as to impede the disease progression and/or improve the efficacy of anticancer therapy [23].

Hypoxia i.e tumor cell oxygen deficiency represents a key micro-environmental stressor governing multiple phenomenon associated with tumor progression such as tumor cell proliferation [24], cell adhesion [25], cell migration [24], tumor neoangiogenesis [26] and metastatic transformation [27]. The recruitment of macrophages to tumor and the plasticity of their responses have recently gathered considerable interest as potential therapeutic target. While macrophages are known to preferentially concentrate in hypoxic areas of tumors [28], the exact mechanism underlying homing of macrophages to hypoxic regions remains elusive. Furthermore effect of hypoxic tumor microenvironment on phenotype switching by macrophages remains less well studied and needs detailed investigation. Experimental evidence demonstrate that macrophages predominantly exhibit an M1-phenotype in the areas of chronic inflammation where tumors commonly develop, while in established tumors, they display a tumor promoting M2 like phonotype [29]. Consequently, it has been hypothesized that the infiltrated macrophages initially have an M1-polarized phenotype; however continued presence in tumor microenvironment polarizes them to M2-skewed TAMs [29]. Hypoxic tumor milieu has been proposed to be the most probable cause of phenotype switching [29]. Consistent with this, the hypoxic area of human endometrial [30], breast [31, 32], prostate [33] and ovarian carcinomas [34] are known to have large congregation of M2 like TAMs. Although the evidence in support of hypoxic microenvironment being instrumental in macrophage recruitment and polarization are gradually mounting, the exact mechanism underlying sequestration of macrophages to hypoxic regions and their subsequent polarization is poorly understood and warrants an in-depth investigation. In the present study we evaluated whether tumor hypoxia in addition to its well described entrapment of macrophages to hypoxic necrotic region could also affect macrophage infiltration and macrophage M2 polarization through an alternate mechanism involving signals emanating from hypoxic tumor cells. Here we suggest a possible mechanism that might contribute towards macrophage infiltration and macrophage M2 polarization with in hypoxic tumor areas. We present data identifying hypoxic tumor cell derived Oncostatin M and Eotaxin as critical regulators of macrophage infiltration and their polarization to M2 skewed macrophages. Besides promoting inflammation, Oncostatin M is known to contribute towards epithelial mesenchymal transition (EMT) [35], breast cancer metastasis [36]. Although both the cytokines have been suggested to promote metastasis, underlying mechanism is not clear. Our study brings forth possible mechanism by way of which Oncostatin M and Eotaxin may promote cancer metastasis. Our results indicate that Oncostatin M and Eotaxin may potentiate breast cancer metastasis by promoting macrophage infiltration and/or M2 polarization. These results may have implications for hindering the M2 polarization of macrophages or better still reprogramming of M2 macrophages to acquire an M1-like phenotype so as to restore anti tumor effects of macrophages which in turn may lead to better patient prognosis and improved response to therapy.

## RESULTS

### Hypoxic breast cancer cells attract macrophages through the release soluble mediators

**Fig 1:**
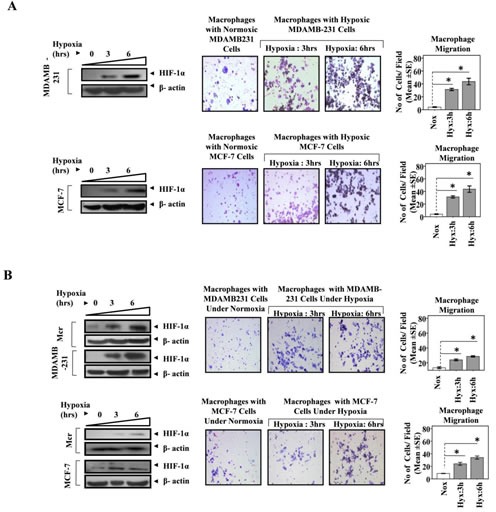
Hypoxia Primed Breast Cancer Cells Chemoattract Macrophages **(A)** MDA-MB-231 and MCF-7 breast cancer cells cultured in lower well of modified boyden chamber were exposed to hypoxic environment for 3and 6 hrs. THP-1 derived macrophages previously cultured on PET cell culture inserts were introduced in upper well. Extent of macrophage migration was evaluated after maintaining the co-cultures for further 24 hrs under standard cell culture conditions. **(B)** Breast cancer cells (MDA-MB-231 and MCF-7) and Macrophage (THP-1 derived macrophages) co-cultures were exposed to hypoxic environment for 3and 6 hrs. Extent of macrophage migration was evaluated after maintaining the co-cultures for further 24 hrs under standard cell culture conditions. Representative western blot data showing hypoxia mediated upregulation of HIF1-α in MDA-MB-231, MCF-7 breast cancer cells and THP-1 derived macrophages as an indication of hypoxic stress. Photomicrographs (100X) depict macrophage chemotaxic towards breast cancer cells as evaluated through Geimsa staining of migrated macrophages. Quantification of macrophage chemotaxis was done by DAPI staining of migrated macrophages followed by counting of nuclei in five different fields of three replica wells. Data presented as Mean±SEM; n=5; Symbols indicate statistical significance at p < 0.05 (*).

Although accumulation of TAMs to hypoxic/necrotic regions of tumor is well established, the underlying mechanisms are unclear. The well reported entrapment of macrophages to hypoxic region could presumably be a contributing factor, but the instruments that in the first place home the macrophages to hypoxic regions are obscure and warrant detailed investigation. We hypothesized that cancer cells in response to hypoxic stress trigger macrophages recruitment. To test this hypothesis we evaluated the transmigration of THP1 cells (Human leukemia monocyte THP1 cell line) derived macrophages towards MDA-MB-231 and MCF-7 human breast cancer cells that were previously exposed to hypoxic conditions for 3 and 6 hrs. The MDA-MB-231 and MCF-7 human breast cancer cells grown in lower well of modified Boyden were exposed to hypoxia for 3 and 6 hrs. That our experimental conditions could successfully elicit hypoxic stress in breast cancer cells was separately verified by assessing the expression profile of HIF1α, an established cellular biomarker of hypoxia (Fig 1a & 1b). The HIF1α expression exhibited a time dependent increase in response to hypoxia. The cells maintained in normoxic conditions served as control. At the end of treatment, the hypoxic breast cancer cells were brought back to normoxic environment. Thereafter THP-1 derived macrophages cultured in 8.0μM PCF cell culture inserts were introduced to the upper well of the modified Boyden chamber. The non-contact co-cultures were then maintained for 24 hrs so as to monitor the migration of macrophages towards hypoxic or normoxic breast cancer cells. Over a period of 24 hrs, the THP-1 derived macrophages exhibited greater migration towards hypoxia primed breast cancer cells than towards normoxic breast cancer cells (Fig. 1A). Quantitative analysis revealed that the extent of macrophage transmigration positively correlated with the duration for which breast cancer cells were exposed to hypoxia. However when co-cultured breast cancer cells and THP1 derived macrophages were simultaneously exposed to hypoxic conditions for similar durations, the macrophages migrated to a much lesser extent (Fig. 1B; Supl. 1). This was in accordance with previous reports indicating impaired migratory potential of macrophages in hypoxic environment [28, 35]. The results indicated that hypoxia primed breast cancer cells release certain soluble factors that promote directional migration of macrophages.

**Fig.2:**
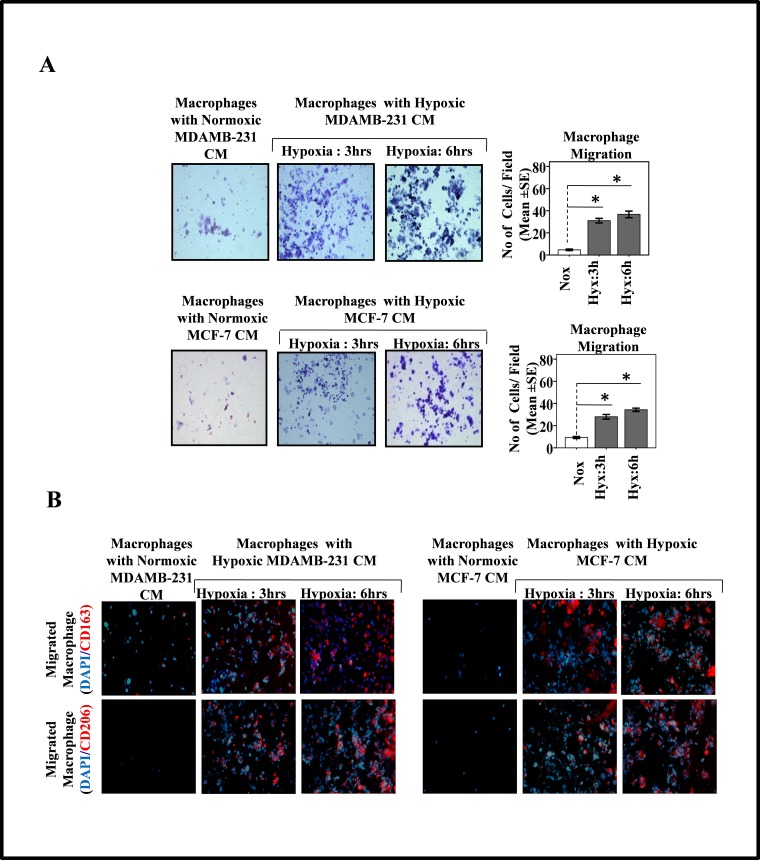
Hypoxia Primed Breast Cancer Cells Release Soluble Mediators to Chemoattract Macrophages and Migrated Macrophages Exhibit M2-polarized phenotype **(A)** THP-1 derived macrophages previously cultured on PET cell culture inserts were introduced in upper well of modified boyden chamber while lower well contained conditioned media from hypoxia primed (3 and 6hrs) breast cancer cells (MDA-MB-231 and MCF-7). Extent of macrophage migration was evaluated after maintaining the cultures for further 24 hrs under standard cell culture conditions. Photomicrographs depicts directional migration of macrophages towards hypoxic breast cancer cells conditioned media as evaluated through Geimsa staining of migrated macrophages followed by bright field microscopy (100X). The Quantification of macrophage migration was done by DAPI staining of migrated macrophages followed by counting of nuclei in five different fields of three replica wells. **(B)** Representative photomicrographs depicting M2-polarization of migrated macrophages as measured through immunocytochemical detection of M2 specific markers viz. CD163 and CD206. Data presented as Mean±SEM; n=5; Symbols indicate statistical significance at p < 0.05 (*).

Previous reports implicate Colony stimulating factor-1 (CSF-1) secreted by breast cancer cells as potent chemoattractant for macrophages [36-38]. Furthermore tumor cell hypoxia is known to regulate CSF-1 expression. While hypoxic microenvironment downregulates CSF-1 expression in several tumors cell lines, the normoxic tumor cells express much upregulated levels of CSF-1. In our experimental setting, the initial exposure of breast cancer cell to hypoxia was followed by their switching to normoxic conditions. This switching from hypoxic to normoxic conditions and further mantainence for 24 hrs was likely to upregulate CSF-1 levels as normoxic tumor cells are known to express much higher levels of CSF-1 than the hypoxic tumor cells. Thus the enhanced macrophage chemotaxis, could also be attributable to normoxia mediated induction and release of CSF-1, rather than signals emanating from hypoxic tumor cells. To rule out this possibility, the ability of hypoxic breast cancer conditioned media (CM) to chemoattract THP-1 derived macrophages was evaluated over a period of 24 hrs, THP-1 derived macrophages migrated towards hypoxic breast cancer CM to much greater extent than towards normoxic breast cancer CM (Fig. 2A). These observations indicated that migration of macrophages towards hypoxic breast cancer cells was indeed due to soluble factors released by breast cancer cells in response to hypoxia and not due to factors released during subsequent normoxic conditions.

**Fig.3:**
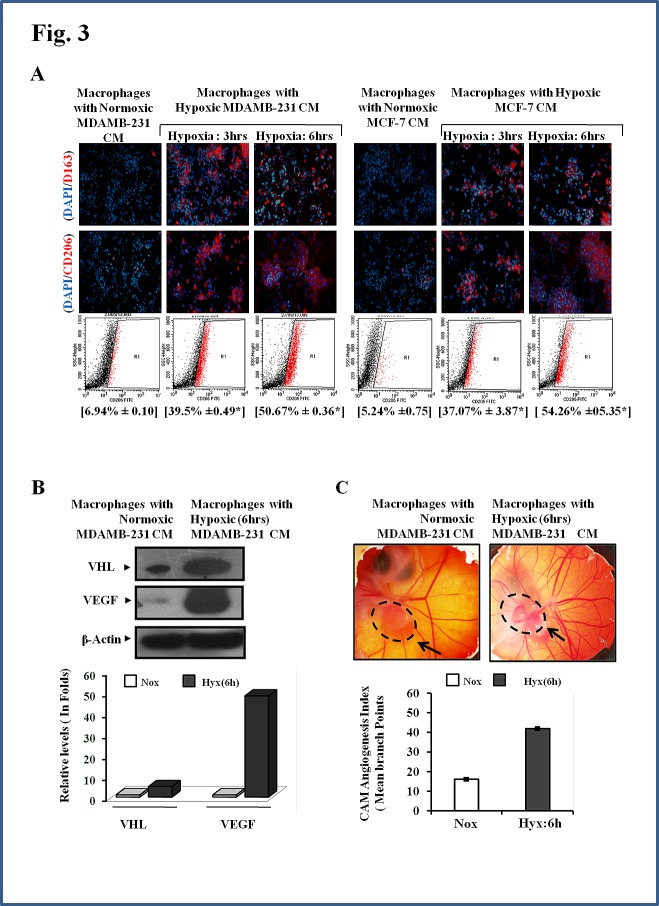
Enhanced M2-polarization of Macrophage with Potentiation of Pro-angiogenic Function by Hypoxic Breast Cancers Cells through the Release of Soluble Mediators THP-1 derived macrophages were incubated with conditioned media from hypoxia primed (3 and 6hrs) breast cancer cells (MDA-MB-231 and MCF-7) CM for 24 hrs, followed by phenotype evaluation using immunocytochemistry and flow cytometry. (A) Representative photomicrographs and flow cytometry data depicting enhanced M2-polarization of THP-1 derived macrophages in presence of hypoxia primed breast cancer cells conditioned media as measured through immunocytochemistry and flow cytometry analysis using Alexa fluor 555 or FITC conjugated anti-CD206 antibody respectively. Values in parenthesis represent mean ± SEM (n=3) of % M2-macrophage count obtained during flow cytometric analysis of three independent experiments. (B) Representative qualitative and quantitative western blot data showing hypoxia primed breast cancer cells conditioned media induced upregulation of key angiogenic regulators viz. Von Hippal-Lindau protein (VHL) and Vascular Endothelial Growth Factor (VEGF) with in macrophages. (C) Representative CAM assay stereozoom micrograph and CAM Angiogenesis index as a measure of angiogenic potential of macrophages incubated with normoxic or hypoxic breast cancer cells CM. Dotted circle represents gelatin sponge graft site.

Tumor associated macrophages exhibit an M2 polarized phenotype. We therefore decided to explore whether the macrophages that migrated towards hypoxic breast cancer CM, too had an M2-skewed phenotype. Presence of M2 specific surface marker viz. CD206 and CD163 was employed as a means to characterize the phenotype of migrated macrophages. Immunocytochemistry studies revealed that the macrophages that selectively migrated towards hypoxic cells CM did express CD206 and CD163 abundantly (Fig. 2B). Since very few macrophages migrated towards normoxic cells CM, their phenotype could not be ascertained clearly. Nonetheless the results pointed towards the possibility of hypoxic breast cell CM causing polarization of macrophages towards an M2 skewed phenotype. In order to ascertain this, we examined the phenotype of THP 1 derived macrophages after incubating them with hypoxic or normoxic breast cancer CM for 24 hrs followed by immunocytochemical and flowcytometric detection of M2 specific markers viz. CD206 and CD163. Results of immunocytochemical analysis revealed that macrophages incubated with normoxic breast cancer cells CM expressed CD206/CD163 minimally (Fig. 3A). On the other hand macrophages grown in hypoxic breast cancer cell CM media expressed the M2 markers much abundantly. The flowcytometric analysis using FITC conjugated CD206 antibody further validated the immunocytochemistry data. Amongst macrophages maintained with normoxic MDA-MB-231 and MCF-7 cells CM, only 6.94% and 5.24 % macrophages expressed CD206 respectively, while 39.5 and 37.07 % macrophages expressed CD206 when maintained in hypoxia (3hrs) primed breast cancer cells (MDA-MB231 and MCF-7) CM, which further escalated to 50.06% and 54.26 % respectively when duration of hypoxia was increased to 6hrs. One of the important functional attribute of M2 polarized macrophage is their ability to promote angiogenesis [9-13]. Thus we decided to evaluate angiogenic potential of macrophages incubated with normoxic or hypoxic breast cancer cell CM. As an indirect measure of angiogenic potential, expression levels of key angiogenesis regulators viz. anti-angiogenic von Hippel-Lindau protein (VHL) and pro-angiogenic Vascular Endothelial Growth Factor (VEGF) were determined. As compared to macrophages incubated with normoxic breast cancer CM, the ones that were incubated with hypoxia (6hrs) primed breast cancer CM exhibited robust (48 folds) up-regulation of VEGF levels (Fig.3B). Paradoxically, the expression level of anti-angiogenic VHL was also enhanced albeit to a much lesser extent (5 folds). Concomitant upregulation of these two factors having mutually opposite function was somewhat confounding. Therefore, chorioallantoic membrane (CAM) assay was carried out as a direct measure of angiogenic potential of macrophages incubated with normoxic or hypoxic breast cancer cell CM. Results revealed that macrophages incubated with culture supernatant of hypoxic breast cancer exhibited much higher angiogenesis index compared to that of macrophages incubated with culture supernatant of normoxic breast cancer cells (Fig. 3C). Results ascertained that hypoxic cells secrete certain soluble mediators in the culture supernatant which not only act as chemotactic factor for surrounding macrophages, but also eventually stimulate them to acquire the pro-tumor M2 polarized phenotype.

### Hypoxic breast cell conditioned media exhibited increased levels of Oncostatin M and Eotaxin

**Fig.4:**
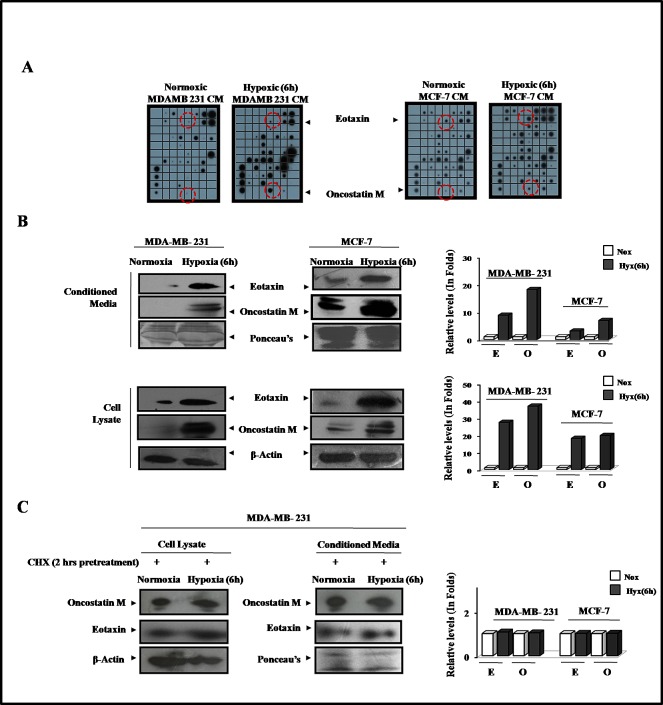
Upregulated Expression and Secretion of Oncostatin M and Eotaxin by Hypoxia Primed Breast Cancer Cells MDA-MB-231 and MCF-7 breast cancer cells were exposed to hypoxic environment for 3and 6 hrs and the culture supernatant was profiled for presence of eighty different cytokines (A) Cytokine profile of hypoxia primed breast cancer cells conditioned media as evaluated through antibody based human cytokine protein membrane array. (B) Representative western blot data (qualitative and quantitative) depicting elevated levels of Eotaxin and OncostatinM in hypoxia primed breast cancer cells conditioned media and cell lysate. (C) Representative western blot data depicting levels of Eotaxin and OncostatinM in the conditioned media and cell lysate of cycloheximide pretreated normoxic or hypoxic breast cancer cells.

Having established that hypoxic cells trigger macrophage recruitment and their M2 polarization, through the release of certain soluble factors, we next decided to identify them. Using antibody based human cytokine protein membrane array we profiled normoxic and hypoxic breast cell CMs for presence of chemokines/cytokines (Suppl. 2). Compared to normoxic cells, the hypoxia primed cells exhibited markedly altered secretome. Comparative analysis of the cytokine profile revealed that exposure to hypoxic conditions elicited the enhanced release of multiple cytokines from cancer cells (Suppl. 3) amongst these the levels of two cytokines viz Oncostatin M and Eotaxin were consistently higher in all the tested hypoxia primed cancer cells CM than that in normoxic cancer cells CM (Fig. 4A).

The findings of cytokine array analysis were further corroborated during western blot analysis of respective conditioned media (Fig. 4B). In order to verify if Oncostatin M and Eotaxin released from hypoxia primed cancer cells were newly synthesized or from the pre-existing cellular repertoire, the cells were pretreated with protein synthesis inhibitor cycloheximide (10ng/ml) 2 hrs prior to hypoxia priming (Fig. 4C). Cycloheximide successfully countered the hypoxia induced upregulation of these cytokines in cells, which in turn was accompanied by inhibition of their release in culture supernatant. Results revealed that hypoxia induces the release of newly synthesized Oncostatin M and Eotaxin rather than the pre-synthesized ones from cancer cells.

### Oncostatin M and Eotaxin are key mediators employed by hypoxic tumor cells to chemoattract macrophages and promote their M2 polarization

**Fig.5:**
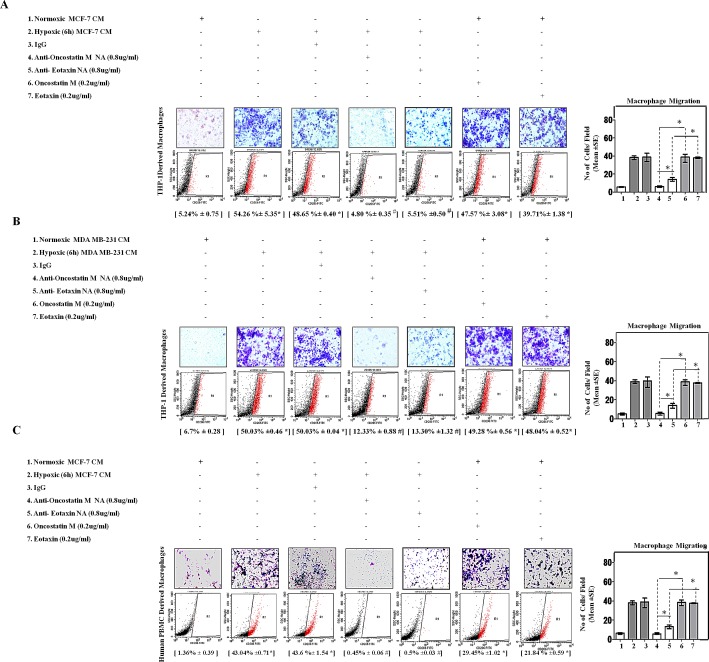
Neutralizing Antibody Mediated Blockade of OncostatinM and Eotaxin Function Prevented Macrophage Chemotaxis and their M2-Polarization THP-1 derived macrophages or Human Peripheral Blood Mononuclear Cells (h-PBMCs) derived macrophages were incubated with conditioned media from hypoxia primed (6hrs) or normoxic breast cancer cells (MCF-7 and MDA-MB-231) CM for 24 hrs in absence or presence of anti-Oncostatin M/anti-Eotaxin neutralizing antibodies or recombinant Oncostatin M/Eotaxin respectively. Thereafter macrophage migration and phenotype switching was evaluated using Geimsa/DAPI staining and flowcytometry respectively. Representative giemsa-staining photomicrographs and Flow cytometry data depicting blockade of directional migration and M2-polarization of THP-1 derived macrophages **(A-B)** or Human Peripheral Blood Mononuclear Cells (h-PBMCs) derived macrophages **(C)** towards hypoxia primed breast cancer cell CM in presence of anti-oncostatin M or Eotaxin neutralizing antibody. Addition of recombinant human Oncostatin M or Eotaxin to normoxic breast cancer cells CM potentiated directional migration and M2-polarization of THP-1 derived macrophages/Human Peripheral Blood Mononuclear Cells (h-PBMCs) derived macrophages. Quantification of macrophage migration chemotaxis was done by DAPI staining of migrated macrophages followed by counting of nuclei in five different fields of three replica wells. Numbers in parenthesis represent % M2-macrophage count obtained during flow cytometric analysis of three replica sets. Data presented as Mean±SEM; n=5; Symbols indicate statistical significance at p < 0.05 (*-#). Differences between values with matching symbol notation are statistically insignificant.

The consistently elevated levels of these two cytokines in the culture supernatant of hypoxic cells led us to question whether these may be the possible mediators employed by hypoxic breast cancer cells to chemoattract and polarize macrophages. In order to address this question the effect of Oncostatin M and Eotaxin blockade was evaluated with respect to macrophage migration and their M2 polarization in presence of hypoxic or normoxic breast cancer CM. Blockade of Oncostatin M and Eotaxin using anti-human Oncostatin M neutralizing antibody and anti-human Eotaxin neutralizing antibody impaired directional migration of macrophages towards hypoxic cells CM (Fig. 5A&B). Flowcytometry analysis indicated that hypoxic breast cancer cell CM induced M2 polarization of macrophages was also markedly impaired in response to neutralizing antibody mediated blockade of Oncostatin M and Eotaxin function. Marked inhibition by both the neutralizing antibodies indicated that macrophage recruitment and polarization is dependent on both the proteins. To corroborate this observation, recombinant oncostatin M and Eotaxin were added to normoxic cells CM and extent of macrophage chemotaxis and polarization was evaluated. Addition of recombinant oncostatin M and Eotaxin to normoxic cancer cell CM resulted in enhanced directional migration of macrophages with a concurrent increase in their polarization towards an M2-skewed phenotype as measured through flowcytometric detection of CD206. Thus in contrast to results with neutralizig antibody, the results with recombinant proteins indicated that both the cytokines may affect macrophage recruitment and polarization independently on their own.

To develop these data further we asked whether human peripheral blood mononuclear cell (huPBMCs) derived macrophages would also exhibit a similar response. Interestingly, huPBMC derived macrophages too exhibited enhanced directional migration towards hypoxia primed breast cancer cells CM with a concurrently increased phenotype switching to M2-polarized macrophages (43.04%) as measured through flow cytometry (Fig. 5C). While neutralizing antibody mediated blockade of Oncostatin M and Eotaxin function impaired hypoxia primed breast cancer cells CM induced macrophage chemotaxis and their M2-polarization, addition of recombinant cytokines to normoxic cells CM caused significant chemotaxis and polarization.

**Fig.6:**
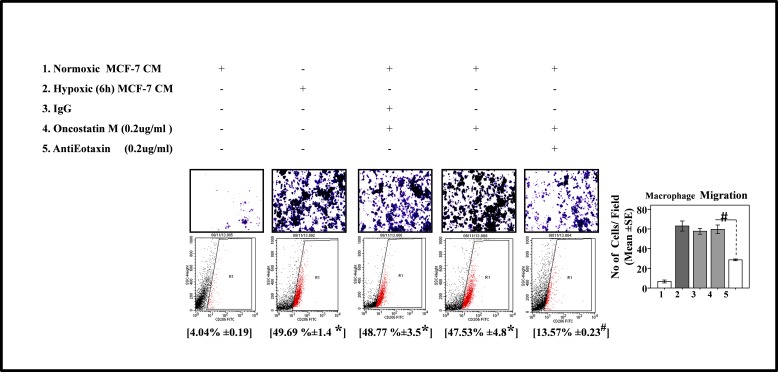
Neutralizing Antibody Mediated Blockade of Eotaxin Prevented Oncostatin M Mediated Macrophage Chemotaxis and their M2-Polarization THP-1 derived macrophages derived macrophages were incubated with conditioned media from hypoxia primed (6hrs) or normoxic breast cancer cells (MDA-MB-231) CM for 24 hrs in presence of recombinant Oncostatin M either alone or in combination with anti-Eotaxin neutralizing antibody or isotype control antibody. Thereafter macrophage migration and phenotype switching was evaluated using Geimsa/DAPI staining and flowcytometry respectively. Representative giemsa-staining photomicrographs and Flow cytometry data depicting blockade of directional migration and M2-polarization of THP-1 derived macrophages. Quantification of macrophage migration chemotaxis was done by DAPI staining of migrated macrophages followed by counting of nuclei in five different fields of three replica wells. Numbers in parenthesis represent % M2-macrophage count obtained during flow cytometric analysis of three replica sets. Data presented as Mean±SEM; n=5; Symbols indicate statistical significance at p < 0.05 (*- #). Differences between values with matching symbol notation are statistically insignificant.

Thus consistently in all the experimental settings, while results with neutralizig antibody indicated that presence of both the proteins is necessary for macrophage recruitment and polarization, paradoxically, the results with recombinant proteins indicated that both the ′cytokines may affect macrophage recruitment and polarization independently on their own. This may be attributable to reported role of Oncostatin M in eotaxin upregulation via PI3K/MAP kinase[39]. Thus we speculated that Oncostatin M apart from being an effector in its own capacity, may also exert its response by via Eotaxin. In order to test this hypothesis, we evaluated the effect of Eotaxin blockade on Oncostatin M induced macrophage chemotaxis and M2-polarization. As expected, addition of Eotaxin neutralizing antibody to normoxic cells CM containing Oncostatin M caused marked impairment of macrophage chemotaxis and M2-polarization as compared to normoxic cells CM containing Oncostatin M alone (Fig.6). These data identified a previously unrecognized role of Oncostatin M and Eotaxin as effetors employed by hypoxic tumor cells to promote macrophage recruitment and their M2 polarization, with Oncostatin M being able to act directly as well as via Eotaxin.

### Human breast cancer specimen exhibited upregulated Oncostatin M and Eotaxin levels in the hypoxic regions with a concurrently elevated M2-macrophages content

**Fig.7:**
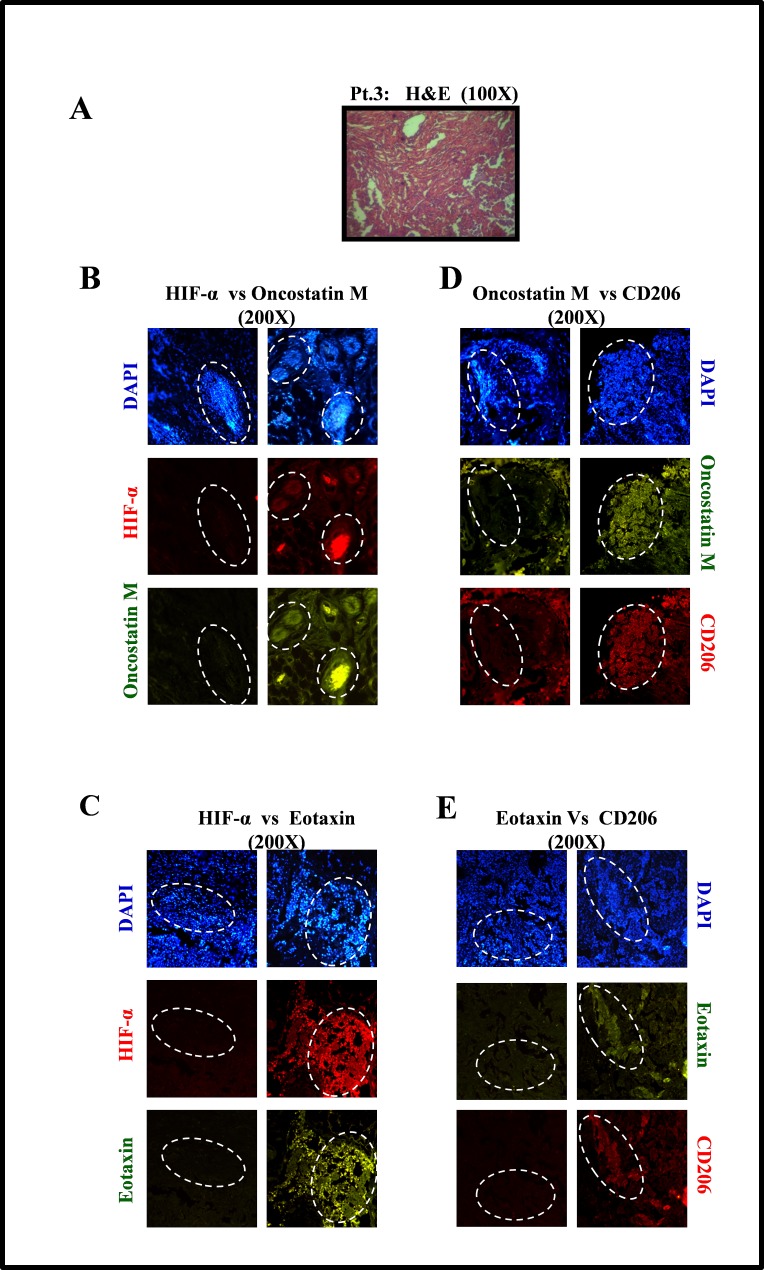
Oncostatin M and Eotaxin Overespression in Hypoxic Regions of Human Breast Cancer Specimen, with Concurrently Upregulated CD206-expressing M2-Macrophages (A) H&E staining revealing distinct tumor architecture in human breast cancer specimens (patient #3) (B) Representative examples of presence of Oncostatin M in hypoxic regions of breast cancer specimen (patient 3) as detected through immunohistochemical staining for hypoxia specific biomarker HIF1α and Oncostatin M. (C) Representative examples of presence of Eotaxin in hypoxic regions of breast cancer specimen as detected through immunohistochemical staining for hypoxia specific biomarker HIF1α and Eotaxin. (D-E) Oncostatin M and Eotaxin positive regions of breast cancer specimen coincided with CD206 enriched regions.

The clinical relevance of this data was evaluated using IHC analysis of human breast cancer specimen. Our *in vitro* results indicated that hypoxic cancer cells exhibited elevated expression and secretion of Oncostatin M and Eotaxin as compared to normoxic cancer cells. To validate this observation we performed immunohistochemical analysis of human breast cancer specimen using HIF-1α as a marker for designating hypoxic regions. Immunohistochemical analysis revealed that Oncostatin M and Eotaxin levels were undetectable in HIF-1α deficient normoxic regions. While the hypoxic regions where HIF-1α was being expressed abundantly, the levels of Oncostatin M and Eotaxin were markedly upregulated (Fig. 7B&C; Suppl. 4&5). Our *in vitro* data indicated that Oncostatin M and Eotaxin accounted for increased macrophage infiltration and M2-polarization. To confirm if the number of M2-like TAMs is higher in Oncostatin M and Eotaxin enriched regions we performed immunohistochemical analysis of human breast cancer specimen using M2-macrophage specific antibody, CD206. Results revealed that M2-macrophage content was much higher in Oncostatin M and Eotaxin enriched regions as compared to that in regions exhibiting diminished levels of these cytokines (Fig. 7 D&E; Suppl. 4&5). Collectively the results led us to concluded that levels of Oncostatin M and Eotaxin were upregulated in the hypoxic area of human breast cancer specimen which in turn coincided with higher number of CD206 expressing M2-macrophages (Suppl.6).

### *In vivo* blockade of OncostatinM or Eotaxin resulted in regression of 4T1 tumor with a concurrent reduction of M2-macrophage content

**Fig.8:**
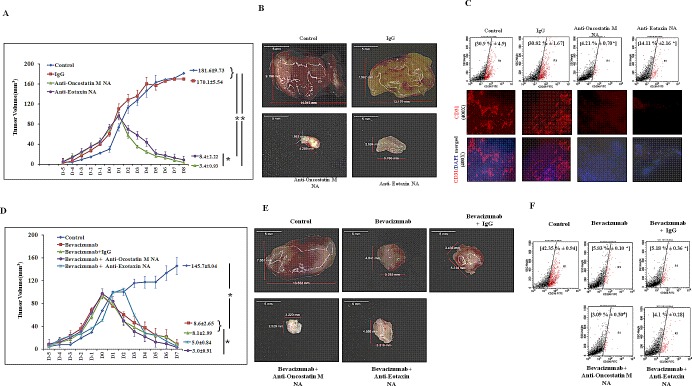
Regression of 4T1 Tumor and Diminished Tumor M2-Macrophage Content Following Neutralizing Antibody Mediated Blockade of Oncostatin M and Eotaxin Function in Syngenic 4T1/BALB/c Mouse Model of Breast Cancer (A) Time course analysis of volume (mean ± SE; n≥5) of control, anti-Oncostatin M or anti-Eotaxin neutralizing antibody or isotype control antibody treated syngenic 4T1/BALB/c subcutaneous tumor specimen. (B) Representative syngenic 4T1/BALB/c subcutaneous tumor specimen at day eight belonging to control group and the groups receiving three courses of anti-Oncostatin M or anti-Eotaxin neutralizing antibody or isotype control antibody IgG injections. (C) Representative flow cytometry data for total number (%) of CD206 positive M2-macrophages and representative immunofluorescence micrograph for presence of endothelial specific marker CD31 in control, anti-Oncostatin M or anti-Eotaxin or control Mouse IgG treated syngenic 4T1/BALB/c tumor specimen. (D) Time course analysis of volume (mean ± SE; n≥5) of syngenic 4T1/BALB/c subcutaneous tumor specimen injected with anti-angiogenic drug Bevacizumab either alone or in combination with anti-Oncostatin M/ anti-Eotaxin/ isotype control antibody. (E) Representative Syngenic 4T1/BALB/c subcutaneous tumor specimen at day seven after it was injected with anti-angiogenic drug Avastin either alone or in combination with anti-Oncostatin M/ anti-Eotaxin/ control Mouse IgG. (F) Representative flow cytometry data for total number (%) of CD206 positive M2 macrophages in syngenic 4T1/BALB/c subcutaneous tumor specimen injected with anti-angiogenic drug Bevacizumab either alone or in combination with anti-Oncostatin M/ anti-Eotaxin/ isotype control antibody. Values in parenthesis represent mean ± SEM (n≥5) of % M2-macrophage count obtained during flow cytometric analysis final tumor specimen at day nine. Symbols indicate statistical significance at p < 0.05 (*).

To determine whether these observation could be replicated in vivo, we employed syngenic 4T1/ BALB/c mouse model of breast cancer. The 4T1 mammary carcinoma is a transplantable tumor cell line that is highly tumorigenic and invasive. Because the model is syngenic in BALB/c mice, and employs animals that have functionally intact immune system, it allows investigators to study role of immune system in tumor progression. Tumor volume analysis revealed that Oncostatin M or Eotaxin blockade resulted in regression of 4T1 tumor (Fig. 8A). Furthermore the Oncostatin M or Eotaxin neutralizing antibody treated 4T1 tumors appeared much less vascularized as compared to control 4T1 tumors (Fig. 8B) as evaluated through immunofluorescence analysis of endoethelial cell specific marker CD31 within 4T1 tumor sections (Suppl.7). Flowcytometry analysis using M2-macrophage specific CD206 antibody revealed that Oncostatin M or Eotaxin blockade resulted in diminished M2-macrophage content with in 4T1 tumor specimen (Fig. 8C).

### Anti-angiogenic agent Bevacizumab exhibited augmented efficacy upon of concomitant blockade of oncostatin M or Eotaxin

The impaired blood supply followed by hypoxia is basis of many anti-angiogenic therapeutics or vascular disruptive therapeutics. TAMs not only promote key processes in tumor progression, they also control response/resistance to therapy by driving reparative mechanisms following radiotherapy or vascular-disruptive therapy. Thus impeding macrophage infiltration and/or their polarization might attenuate commencement of reparative cascade leading to improved efficacy of vascular-disruptive therapy. In order to ascertain this we evaluated the effect of Oncostatin M or Eotaxin blockade during therapy with anti-angiogenic agent Bevacizumab. Tumor volume data revealed that neutralizing antibody mediated concomitant blockade of Oncostatin M or Eotaxin function resulted in better tumor regression as compared to monotherapy with Bevacizumab alone. Here again the improved efficacy could be attributable to impaired macrophage infiltration and/or their polarization as the tumors injected with Bevacizumab and Oncostatin M/Eotaxin neutralizing antibodies exhibited much reduced M2-polarized macrophage content as compared to tumors injected with Bevacizumab alone or Bevacizumab with isotype control antibody.

## DISCUSSION

Solid tumors do not grow beyond 2-3 mm^3^ due to hypoxia [38]. It is a common feature of tumor microenvironment and occurs very early during tumor development due to rapid proliferation of transformed cells [40, 41]. To sustain growth and survival in hostile microenvironment the rapidly dividing tumor cells must overcome hypoxia and lack of nutrients by potentiating tumor neo- angiogenesis [42]. A feat that is achieved by hypoxic tumor cells not completely on their own but by drawing an active support from surrounding stromal cells particularly TAMs [23]. TAMs are resident macrophages that in response to tumor microenvironmental cues undergo phenotype switching to acquire a pro-tumor M2-polarized phenotype and promote tumor progression by potentiating angiogenesis and metastasis [43]. They preferentially accumulate in hypoxic/necrotic regions of tumor and their presence in high numbers has strong association with poor patient prognosis [28, 6]. Consequently, TAMs have emerged as potential targets for anticancer immunotherapy. It has been hypothesized that hindering macrophage infiltration and/or preventing their polarization toward a pro-tumor M2-phenotype, or better still reprogramming the M2-like TAMs towards M1-subtype may effectively counteract tumor progression [43]. However successful translation of these approaches into effective therapeutic strategy requires better characterization of tumor microenvironment derived signals that regulate macrophage recruitment and their polarization, so that the targets/molecular mechanisms amenable to therapeutic intervention may be identified. Furthermore owing to hypoxic milieu being a persistent feature of tumor microenvironment and a major contributor to malignancy and treatment resistance, understanding hypoxic tumor cell responses vis-a-vis macrophage homing and phenotype switching is of paramount significance.

**Fig 9:**
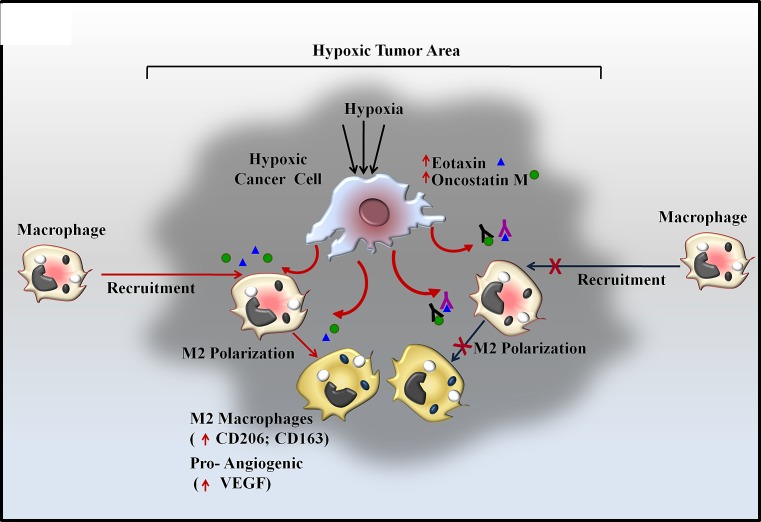
Schematic Representation of Oncosatin M and Eotaxin mediated Recruitment of TAMs and subsequent M2 polarization in Hypoxic Tumor area

In the present study we demonstrated a previously unknown cascade of macrophage homing towards hypoxic tumor cells. It is well documented that upon encountering hypoxic/necrotic environment, macrophages undergo a migratory arrest leading to their entrapment in hypoxic/necrotic regions; however mechanisms accounting for their homing to hypoxic regions are not clear. We demonstrated that even in absence of prior direct exposure to hypoxic conditions, the macrophages do exhibit directional migration towards hypoxic tumor cells. Furthermore the hypoxic tumor cells triggered M2-polarization of THP-1 derived macrophages and imparted them a pro-angiogenic phenotype (Fig.9).

Using immunocytochemical and flowcytometry analysis for detecting M2-macrophage specific markers, we have successfully demonstrated that hypoxic tumor cells trigger M2-polarization of THP-1 derived macrophages. To evaluate whether in the interim, the macrophages co-incubated with hypoxic breast cancer cell CM also acquired a pro-angiogenic phenotype, the expression levels of key angiogenic regulators viz. VHL (anti-angiogenic) and VEGF (pro-angiogenic) were evaluated. We observed upregulation of both these factors albeit to a variable degree. While VEGF exhibited robust increase, the extent of increase was much lesser for VHL. Since VHL is known to downregulate VEGF levels via HIF1α -degradation [44], therefore upregulation of VHL should normally result in VEGF downregulation. On the contrary we observed simultaneous upregulation of both these factors in our study which was somewhat confounding. Possible explanation for this finding could be the fact that VEGF expression is not regulated exclusively in an HIF-1α dependent manner. There are multiple HIF-1α independent pathways of VEGF and angiogenesis regulation, such as HIF independent induction of VEGF by PGC-1α [45] and Akt1 mediated induction of VEGF in an Sp1 dependent manner [46]. Being independent of HIF-1, these pathways may not be regulatable by VHL as such, resulting in enhanced VEGF levels despite potentiated VHL levels. This is particularly plausible in case of cells growing in normoxic milieu where canonical hypoxia response pathway will be minimally active and thus HIF1-α expression may be at basal level. We studied VHL and VEGF levels in macrophages incubated with culture supernatant of hypoxia primed cancer cells and not in hypoxic cancer cells themselves. Since these macrophages never encountered hypoxia and remained in normoxic conditions throughout, they will express HIF1-α only at basal levels. The VEGF levels in these cells will be most likely regulated through HIF-1α independent pathway therefore will be insensitive to VHL levels.

Since there was simultaneous upregulation of anti-angiogenic VHL and pro-angiogenic VEGF, CAM assay was employed as a means to directly asses the angiogenic potential of macrophages incubated with culture supernatant of normoxic or hypoxic breast cancer cells. Exhibition of much higher angiogenesis index by macrophages incubated with culture supernatant of hypoxic breast cancer cells not only demonstrated that hypoxic cancer cells confered pro-angiogenic properties to macrophages, but it also established that pro-angiogenic VEGF overwhelmed the anti-angiogenic effect of VHL. This could be attributable to the fact that anti-angiogenic VHL was upregulated only marginally, while upregulation of pro-angiogenic VEGF was much more substantial.

The enhanced directional migration of macrophages towards hypoxic tumor cells and their polarization to M2-like macrophages was attributable to hypoxic tumor cell derived Eotaxin and Oncostatin M. Hypoxic tumor cells exhibited upregulated intracellular levels of Eotaxin and Oncostatin M, which in turn was accompanied by their enhanced release in the culture supernatant. Interestingly, protein synthesis inhibitor cycloheximide could suppress the release of Oncostatin M and Eotaxin. This demonstrated that release of these cytokines was essentially dependent on their *denovo* synthesis and not on the pre-existing repertoire. Neutralizing antibody mediated blockade of both the cytokines impaired macrophage chemotaxis as well as their M2-polarization indicating that both of them were necessary for the phenomenon to take place. On the contrary, either of recombinant protein when added to normoxic breast cancer cell CM could affect macrophage chemotaxis. This could be attributable to the fact that Oncostatin M through interaction with its cognate receptor induces Eotaxin expression. Thus apart from being an effector on its own, it may also exert response by modulating Eotaxin expression, a possibility that appeared plausible owing to our observation of complete impairment of macrophage chemotaxis and polarization only upon Oncostatin M blockade and not following Eotaxin blockade. Interestingly, ability of Eotaxin neutralizing antibody to partially impair Oncostatin M induced macrophage chemotaxis and polarization substantiated this possibility. Human PMBCs derived macrophages exhibited similar response thereby establishing the physiological relevance of our findings with respect to humans. In agreement with this IHC analysis of human breast cancer specimen also revealed upregulated levels of Oncostatin M and Eotaxin in the hypoxic area which in turn coincided with higher number of CD206 expressing M2-macrophages. Thus our results clearly demonstrate the role of Eotaxin and Oncostatin M as key mediators being released by hypoxic breast cancer cells for chemoattracting and polarizing macrophages towards a M2-skewed phenotype with a pro-angiogenic function. Our findings unravel a hitherto unknown cascade initiated by hypoxic breast cancer cells whereby in an attempt to adapt to the hypoxic tumor microenvironment and ensure their continued survival and sustenance they modulate the overall balance of local TAMs subpopulation. By chemoattracting macrophages and inducing them for phenotype switching to pro tumor M2-macrophages, hypoxic tumor may effectively convert a hostile microenvironment into a survival conducive microenvironment. Results of our neutralizing antibodies based *in vitro* experiments suggest a means to target these events for retarding tumor progression. In agreement with this the *in vivo* studies employing syngenic mice model of breast cancer revealed that administration of anti-Eotaxin or anti-Oncostatin M antibody resulted in significant reduction of tumor volume as compared to mock control or IgG injected mice. Furthermore the anti-Eotaxin or anti-Oncostatin M antibody injected tumors were less vascular. This may be attributable to attenuated M2 polarization of macrophages into pro-angiogenic macrophages in presence of anti-Eotaxin or anti-Oncostatin M antibodies. Thus we propose that inhibiting Eotaxin and Oncostatin M functions using specific antibodies may represent an innovative approach to control macrophage homing and their M2 polarization and resultant tumor progression.

Besides being a key microenvironmental determinant of tumor progression, Hypoxia also remains key factor governing response and/or resistance to therapy [23]. It is the key underlying mechanism for many anti cancer therapeutics such as anti-angiogenic agents or vascular–disrupting agents [47]. Anti-angiogenic or VDA therapeutics act via selective disruption of tumor associated vasculature leading to vessel collapse, reduced blood flow, tumor hypoxia and secondary tumor cell death. However the efficacy of such treatment is generally compromised by TAMs driven reparative mechanisms. For instance, blocking pro-angiogenic factor angiopoitin-2 leads to angiogenesis inhibition and tumor hypoxia, however subsequent enhancement in recruitment of MRC1^+^ TEMs limits the efficacy of ANG-2 blockade [48, 49]. Consequently, simultaneous targeting of TAMs mechanisms has been suggested as an effective means to increase the efficacy of anti-angiogenic therapy. Treatment of tumor with CA-4-P, a potent VDA initially caused selective destruction of tumor blood vessels resulting in hypoxia and necrosis. However VDA-associated hypoxia eventually also resulted in elevated CXCL12 levels and increased TEM infiltration in mammary tumor models. Blocking TEM recruitment either using CXCR4 antagonist plerixafor or by genetic TEM depletion markedly enhanced the efficacy of CA-4-P treatment in subcutaneous N202 (Neu+) mammary/carcinoma model [50]. Furthermore Depletion of TAMs by clodronate-loaded liposomes augmented the inhibitory effects of sorafenib on tumor angiogenesis, growth and metastasis in hepatocellular carcinoma xenograft models [51]. TAM depletion by clodrolip or a CSF-1R inhibitor increased the anti-angiogenic and anti-tumor effects of VEGF-VEGFR2 antibodies [52]. Collectively these data suggest that concomitant targeting of macrophages may not only improve the efficacy of anti-angiogenic drugs but may also hinder the development of resistance to anti-angiogenic therapeutics. In line with this our studies employing syngenic mice model of breast cancer reveal that simultaneous administration of anti-Eotaxin or anti-Oncostatin M antibody greatly increases the efficacy of anti-angiogenic drug Bevacizumab. Targeting macrophages using anti-Eotaxin or anti-Oncostatin M antibody may be more advantageous since this strategy not only hinders macrophage recruitment but also hinders their polarization into a pro-tumor M2 macrophage subpopulation, thus minimizing the chances of development of resistance due to commencement of M2-Like TAMs mediated pro-angiogenic cascades. These findings have important experimental and clinical implication as they point towards a novel combination therapy whereby utilizing antibody mediated selective inhibition of Eotaxin and Oncostatin-M may improve the efficacy of anti-angiogenic agents such as Bevacizumab. In conclusion we have demonstrated hypoxic breast cancer cells selectively chemoattract and polarize macrophages into M2 subtype. Our data demonstrate that hypoxic tumor cells control directional migration and M2 polarization of macrophages via two chemokine/cytokines viz. Eotaxin and Oncostatin M (Fig.9) Our study establish these two chemokines as novel targets for devising novel anticancer therapy whereby targeting them using specific antibody may not only retard tumor progression on its own, but when administered in combination it may augment the efficacy of anti-angiogenic therapy, particularly in tumor that are refractory or develop resistance to anti-angiogenesis therapeutics. Detailed time course analysis of cancer cell proliferation under hypoxic condition vis a vis their ability of modulate macrophage response is an essential prerequisite before these experimental approaches could be translated to effective therapeutic regimen, nonetheless, our study establishes these chemokines as two of the key mediators of macrophage responses during tumor progression and as potential targets for devising newer anti-cancer regimen with better therapeutic efficacy.

## MATERIAL AND METHODS

### Anti bodies and Reagents

Anti-VHL polyclonal antibody and monoclonal antibodies against VEGF, CD206, CD163 and β-Actin were purchased from Santacruz (USA). Recombinant human OncostatinM, recombinant human Eotaxin, anti-Oncostatin M neutralizing antibody and anti-Eotaxin Neutralizing antibodies were procured from R&D Systems (USA). RayBio human cytokine antibody array (5) was procured from Ray Biotech (USA). FITC conjugate of anti-CD206 antibody was procured from BD Biosciences. Alexafluor 488 and Alexafluor 555 conjugates of anti-mouse and anti-rabbit IgG were procured from invitrogen (USA). HRP conjugates of rabbit and mouse IgG were purchased from Cell Signaling Technology (USA) 8μm polycarbonate (PCF) cell culture inserts were purchased from Millipore (USA). Human plasma fibronectin and Phorbol 12-myristate 13-acetate (PMA) were procured from GIBCO-Invitrogen Corporation (USA) and Calbiochem (USA) Respectively.

### Cell Culture and *In Vitro* Differentiation

Human leukemia monocyte THP-1 cells, human mammary cancer-derived (MCF-7 cells and MDA-MB-231) cells were procured from ATCC. Cells were maintained in RPMI 1640 or DMEM respectively supplemented with 10% FBS and 100μg/ml penicillin, 0.25 μg/ml amphotericin B and 100μg/ml streptomycin in a humidified atmosphere (95% humidity) at 37°C and 5% CO_2_. Human peripheral blood mononuclear cells (PBMCs) were isolated from freshly prepared human buffy coats obtained from blood samples of healthy human volunteers after informed consent. THP-1 cells were differentiated to macrophages according to Dockrell et al [53]. The differentiation was initiated by adding 30nM phorbol 12-myristate 13-acetate (PMA) to the cells. After 3 days cells were switched to PMA free media for further 5 days so as to ensure maximal differentiation. Human PBMCs were allowed to differentiate into resting macrophages after 7 days of culture in RPM1 1640 [54]. Differentiation was ascertained by evaluating the expression of macrophage specific markers viz, CD16 and Myeloid Cell Leukemia sequence-1(Mcl-1).

### Hypoxia Treatment

Cells were exposed to hypoxic environment within the hypoxia chamber (Stem cell technologies, USA) maintained at low oxygen tension (1% O_2,_ 5% CO_2_ and 94% N_2_). The treatment was initiated by introducing the culture in the hypoxia chamber and replacing the existing culture medium with deoxygenated RPMI 1640/DMEM. Deoxygenated medium was prepared prior to each experiment by equilibrating the medium with a hypoxic gas mixture containing 1% O_2,_ 5% CO_2_ and 94% N_2_ at 37°C. The oxygen concentration in the hypoxic chamber and the exposure medium was monitored by using an oxygen indicator (Forma Scientific, Marietta, OH).

### Migration assays

THP-1 derived and human PBMCs derived macrophages were seeded onto fibronectin coated 8μm PCF transwell cell culture inserts. For evaluation of migratory potential, the transwell cell culture inserts housing macrophages were introduced to upper well of the modified chamber, while the lower wells contained hypoxic/normoxic breast cancer cells or the respective conditioned media. The co-cultures were incubated for 24 hours under standard cell culture environment (5% CO_2_, 37°C). After 24 hrs the non migrated cells were wiped off from top of the insert membrane using sterile cotton swabs. Migrated cells adhered to the underside of membrane were fixed and stained with Giemsa nuclear stain for qualitative visualization of migration using Eclipse BOi-0.90 Dry Bright-Field Microscope (Nikon, Japan). The extent of migration was quantified by DAPI nuclear staining followed by counting of nuclei in five different fields of three replica sets using Leica DCF450C fluorescence microscope.

### Fluorescence immunocytochemistry and Flow Cytomrtry

The presence of M2-macrophage specific cell surface markers was detected using fluorescence immunocytochemistry and flowcytometry. For fluorescence immunocytochemical detection of M2-polarized macrophage, the culture supernatant of control and experimental macrophage cultures (grown in sterile coverslips or 8μm PCF cell culture inserts) was removed and cells were washed twice with DPBS, followed by fixation with 3.7% paraformaldehyde at 37°C. After washing with DPBS thrice the specimen were blocked with 5% BSA for 1 hr. Thereafter cells were incubated overnight with anti-human CD206 or anti-human CD163 mouse antibodies (1:100) at 4°C. Specimens were then incubated with Alexa fluor 555 conjugated anti- mice IgG (1:100) for 1hr. Finally cells were mounted in prolong gold antifade-DAPI aqueous mounting media and visualized (200X) using Leica DCF 450C florescence microscope. For flow cytometry based detection of CD206 positive M2-macrophges, the control and experimental macrophage cultures were fixed with 3.7% paraformaldehyde for 20 min at 37°C. Thereafter cells were permealized with 0.5% TritonX for 5 minutes, washed with PBS twice and harvested for flow cytometry. Cells were suspended in PBS and incubated with FITC conjugated anti- CD206 antibody for 1 hrs at 4°C. For flow cytometry based detection of CD206 positive M2-macrophages content of 4T1 tumors, the excised tumors were minced finely in sterile PBS and strained through 70μm mesh. The suspension was centrifuged at 500xg for 5 min. Thereafter pellet was suspended in collagenase enzyme solution (1mg/ml in PBS) and allowed to digest for 10 min at 37°C. Cells were suspended in PBS and incubated with FITC conjugated anti- CD206 antibody for 1 hrs at 4°C. Finally, 10,000 viable cells was analyzed by flow cytometry using FACS Calibur flow cytometer (BD Biosciences, USA)

### Chick Chorioallantoic Membrane assay

To detect *in vivo* angiogenesis, we conducted Chick Chorioallantoic Membrane (CAM) assays. Sterile gelatin sponge (4X4mm) soaked in conditioned media of hypoxic or a normoxic MCF-7/MDA-MB-231 cell was implanted onto the CAM at day 8 of fertilization. At day 12, CAMs were fixed with 10% formalin; the neovasculature was examined and photographed. Angiogenesis was quantified by counting the blood vessel branch points under a M205 FA Leica stereozoom microscope.

### Real-time PCR

Total RNA was isolated with TRI reagent (Molecular Research Center), and cDNA was obtained from 2μg of total RNA using High Capacity cDNA Reverse Transcription Kit (Applied Biosystems, USA). Quantitiative PCR was performed on a Light Cycler 480 System (Roche) 96-well plates using SYBR Green qPCR Master Mix (Invitrogen) in accordance with manufacturer's protocol. Data were analyzed using the Roche LightCycler 480 software (Version 1.5). Cp and Ct were calculated by the Second Derivate Maximum Method. The amount of the target mRNA was examined and normalized to the β-actin gene mRNA. The relative expression ratio of a target gene was calculated as described by Pfaffi [55]. Results represent data from three separate experiments. Details of primers and there neucleotide sequences are represented in Suppl. 5

### Chemokine/cytokines profiling of cell culture supernatant

Eighty different cytokines was detected for their presence in culture supernatant of hypoxic or normoxic breast cancer cells using antibody-based human cytokine array (Ray Biotech, USA) according to manufacturer's protocol. Salient findings were further corroborated through western blott analysis of concentrated culture supernatant (10X) or cell lysate.

### Immunoblotting

Cells were lysed in radioimmunoprecipitation assay (RIPA) buffer containing protease and phosphatase inhibitors (1mM phenylmethylsulfonyl fluoride, 10mg/ml aprotinin, and 10mg/ml leupeptin, 10μM sodium orthovanadate). Thereafter lysates were centrifuged at 13,000 × g at 4°C for 30 min. Immunoblotting of cell culture supernatant was performed after appropriately concentrating it using Amicon Ultra centrifugal filters ultracel-10K(Amicon, USA). Protein content in the samples was determined using Folin Lowry's method [56]. Cell culture supernatant or cell lysate supernatants equivalent to 100 μg of protein were resolved through 12% SDS-PAGE and were transferred to PVDF (Millipore, USA) membranes. After blocking with 5% BSA in PBS containing 0.2% Tween-20, PVDF membranes were incubated at 4°C overnight with the different antibodies. Blots were then incubated for 1 h at room temperature with horseradish peroxidase-conjugated secondary antibody (1:2000) and the peroxidase activity was analyzed with the ECL chemiluminiscence substrate system (USA). The expression level of various proteins was quantified by measuring the intensity respective bands using ImageJ software (ImageJ, National Institute of Health, Bethesda, MD). Intensity of loading control i.e β-actin bands (in cell lysate) ponceau's (conditioned media) was used for normalizing the expression levels.

### H&E staining and Immunohistochemistry

H&E staining and Immunohistochemical detection of HIF1-α, Eotaxin, Oncostastin M CD-206 and CD31 was performed on formalin-fixed, paraffin- embedded tissue sections (8μm). Briefly, the deparaffinized and rehydrated tissue sections were incubated overnight with respective primary antibodies viz. anti-HIF1-α mouse monoclonal antibody (1:100), anti-Eotaxin goat polyclonal antibody (1:100), anti-Oncostatin M goat polyclonal antibody (1:100), anti-CD-206 mouse monoclonal antibody (1:100) and anti-CD31 rabbit monoclonal antibody (1:100) after heat-induced epitope retrieval (HIER) with citrate buffer (pH 6.0). Thereafter samples were incubated for 1 hour with Alexa-fluor 488 conjugated donkey anti-goat IgG (1:200) or Alexa fluor 555 conjugated donkey anti-mouse IgG (1:200) or Alexa Fluor 555 conjugated donkey anti-rabbit IgG (1:200). Sections were mounted in Prolong gold antifade-DAPI aqueous mounting medium (Invitrogen USA) and visualized (200X) using Leica DCF450C bright field/fluorescence microscope. The presence of various antigens was quantified by measuring the intensity respective immunocomplexes using ImageJ software (ImageJ, National Institute of Health, Bethesda, MD). Tumor architecture was visualized by examining an additional hematoxylin and eosin stained section of each specimen (200x) using Eclipse BOi-0.90 Dry Bright-Field Microscope (Nikon, Japan)/ Leica DCF450C bright field/fluorescence microscope.

### Animals

All procedures with mice were done under an Institutional Animal Care and Use Committee-approved protocol (#IAEC/2013/44). Female BALB/c mice of 8 weeks age were procured from institutional laboratory animal facility. Mice were housed in polypropylene cages in a group of five/cage. They were maintained on pellet diet, water *ad libitum*, and regular alternate cycles of 12 hours light and darkness. Prior to tumor initiation, animals were acclimatized for 7 days.

### *In vivo* tumor experiment

4T1/BALB/c tumors were initiated by injecting 1×10^6^ viable 4T1 cells subcutaneously in the pre-shaved skin of the back of the BALB/c mice. After two days the animals were boosted by injecting another 1x10^6^ viable 4T1 cells at the initial injection site. The growth of tumor was monitored throughout the experiment with tumor size being measured daily using vernier calipers and represented in terms of tumor volume [=4/3π X(Long axis/2) X(Short axis/2)]. After the tumor attained a size of 6-9 mm^3^, the tumor bearing mice were randomized into four groups based on tumor volume with each group comprising of three-five mice. After observing tumor volume for four days, mice were treated with anti-Oncostatin M/anti-Eotaxin neutralizing antibody or isotype control antibody at tumor site on day 0, 3 and 6 at the dose of 5ug/mice. An additional group receiving equal amount of vehicle (PBS) served as control. At the end of experiment i.e. at day 7 or 8, animals were euthanatized under anesthesia and tumor was excised. The tumor samples were visualized and photographed using Leica M205FA stereo zoom microscope (magnification: 10x; Optical zoom: 0.88) and processed further for flow immunohistochemistry.

### Samples from human breast cancer

Human breast tumor specimens were collected after informed consent from fourteen patients who underwent surgery for breast cancer at King George Medical University, Lucknow (India) The ethics committee at King George Medical University, Lucknow (India) approved the study protocol (#ECM IIB/P17), which followed the Declaration of Helsinki.

### Statistical Analysis

*S*tatistical evaluations were done using SPSS 17.0. Experimental data was analyzed using one way anova followed by Bonferroni post hoc test. For all tests, the level of significance was set at p<0.05. Error bars throughout the figures indicate standard error.

## SUPPLEMANTAL INFORMATION FIGURES AND TABLES


