# Supplementary material for: Macrophages are recruited to hypoxic tumor areas and acquire a Pro-Angiogenic M2-Polarized phenotype via hypoxic cancer cell derived cytokines Oncostatin M and Eotaxin
Source: Oncotarget. 2014 Jun 17;5(14):5350–68. doi: 10.18632/oncotarget.2110 (PMC4170629; doi:10.18632/oncotarget.2110)
Supplement: Supplementary file 1 [file oncotarget-05-5350-s001.pdf]

# Macrophages are recruited to hypoxic tumor areas and acquire a Pro-Angiogenic M2-Polarized phenotype via hypoxic cancer cell derived cytokines Oncostatin M and Eotaxin

## Supplemantal Information

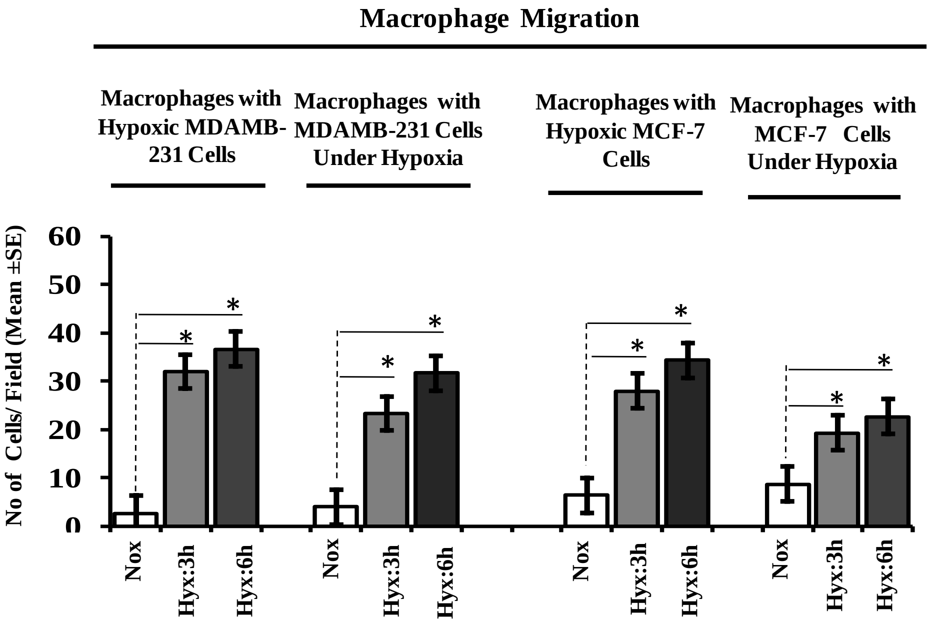


**Suppl.1: Comparison Chemotaxic Data for Macrophage Co-Culture Hypoxic Cancer Cells Vs Macrophage/Cancer Cells Co-Culture Subject to Hypoxia**

## Suppl.2: RayBio Human Cytokine Antibody Array (5) MAP

| | 1 | 2 | 3 | 4 | 5 | 6 | 7 | 8 |
|---|---|---|---|---|---|---|---|---|
| A | GRO-α | IL-10 | MIP-1β | Angiogenin | Eotaxin-3 | IGFBP-2 | NT-3 | Pos |
| B | GRO | IL-8 | MIG | IGF-1 | Eotaxin-2 | IGFBP-1 | NAP-2 | Pos |
| C | GM-CSF | IL-7 | MDC | EGF | Eotaxin | HGF | MIP-3α | TIMP-2 |
| D | GCSF | IL-6 | MCSF | TNF-β | Ckβ8-1 | GDNF | MIF | TIMP-1 |
| E | ENA-78 | IL-5 | MCP-3 | TNF-α | BLC | GCP-2 | MCP-4 | TGF-β3 |
| F | Neg | IL-4 | MCP-2 | TGF-β1 | BDNF | Fractalkine | LIGHT | TGF-β2 |
| G | Neg | IL-3 | MCP-1 | TARC | Leptin | Flt-3 Ligand | LIF | PIGF |
| H | Pos | IL-2 | IFN-ϒ | SDF-1 | PDGF-BB | FGF-9 | IP-10 | PARC |
| I | Pos | IL-1β | IL-15 | SCF | VEGF | FGF-7 | IL-16 | Osteoprotegerin |
| J | Pos | IL-1α | Il-13 | RANTES | Thrombopoietin | FGF-6 | IGFBP-4 | Osteopontin |
| K | Pos | I-309 | IL-12p40p70 | MIP-1δ | OncostatinM | FGF-4 | IGFBP-3 | NT-4 |

| No. | MCF-7 | MDAMB-231 |
|---|---|---|
| 1. | OncostatinM | OncostatinM |
| 2. | - | RANTES |
| 3. | FGF-6 | - |
| 4. | VEGF | VEGF |
| 5. | - | IL-3 |
| 6. | - | MCP-1 |
| 7. | - | TAR-C |
| 8. | Eotaxin | Eotaxin |
| 9. | TNF-β | TNF-β |
| 10. | Eotaxin-2 | - |
| 11. | - | MIP-1β |
| 12. | IL-10 | - |
| 13. | - | Leptin |

**Suppl. 3: List of Human Cytokines Upregulated in Hypoxic Tumour Cell Conditioned Media in Comparison to Normoxic Tumour Cell Conditioned Media**

**Correlation between**

**HIF-1α Vs Oncostatin M**

Total no. of field Evaluated: 74

| | | | | |
|---|---|---|---|---|
| Level of HIF 1-α | High: 41 | | Low /UD: 33 | |
| Level of Oncostatin M | High: 41 | Low/UD: 0 | High: 2 | Low/**UD**: 31 |

Chi square test with (yates corrected) Significant ($p < .001$) association between HIF-1α and Oncostatin M

**Correlation between**

**Oncostatin M Vs CD206**

Total no. of field Evaluated: 60

| | | | | |
|---|---|---|---|---|
| Level of Oncostatin M | High: 33 | | Low /UD: 27 | |
| Level of CD206 | High: 33 | Low/UD: 0 | High: 1 | Low/UD: 26 |

Chi square test with (yates corrected) Significant ($p < .001$) association between Oncostatin M and CD206

**Correlation between**

**HIF-1α Vs Eotaxin**

Total no. of field Evaluated: 65

| | | | | |
|---|---|---|---|---|
| Level of HIF 1-α | High: 36 | | Low /UD: 29 | |
| Level of Eotaxin | High: 36 | Low/UD: 0 | High: 2 | Low/UD: 27 |

Chi square test with (yates corrected) Significant ($p < .001$) association between HIF-1α and Eotaxin

**Correlation between**

**Eotaxin Vs CD206**

Total no. of field Evaluated: 54

| | | | | |
|---|---|---|---|---|
| Level of Eotaxin | High: 31 | | Low /UD: 23 | |
| Level of CD206 | High: 29 | Low/UD: 2 | High: 1 | Low/UD: 22 |

Chi square test with (yates corrected) Significant ($p < .001$) association between Eotaxin and CD206

**Numbers represent fields showing presence of indicated antigen evaluated; UD =Undetectable level.**

## Suppl.4: Collective summary of Immunohistochemical Observations of all (14) the Human Breast Cancer Specimen

## Patient wise correlation between HIF-1α Vs Oncostatin M

**Patient No. 3**

Total no. of field Evaluated: 11

| | | | | |
|---|---|---|---|---|
| Level of HIF 1-α | High: | | Low /UD: | |
| | 7 | | 4 | |
| Level of Oncostatin M | High: | Low/UD: | High: | Low/UD: |
| | 7 | 0 | 0 | 4 |

**Patient No. 25**

Total no. of field Evaluated:4

| | | | | |
|---|---|---|---|---|
| Level of HIF 1-α | High: | | Low /UD: | |
| | 2 | | 2 | |
| Level of Oncostatin M | High: | Low/UD: | High: | Low/UD: |
| | 2 | 0 | 0 | 2 |

**Patient No. 28**

Total no. of field Evaluated:4

| | | | | |
|---|---|---|---|---|
| Level of HIF 1-α | High: | | Low /UD: | |
| | 4 | | 0 | |
| Level of Oncostatin M | High: | Low/UD: | High: | Low/UD: |
| | 4 | 0 | 0 | 0 |

**Patient No. 41**

Total no. of field Evaluated:5

| | | | | |
|---|---|---|---|---|
| Level of HIF 1-α | High: | | Low /UD: | |
| | 2 | | 3 | |
| Level of Oncostatin M | High: | Low/UD: | High: | Low/UD: |
| | 2 | 0 | 0 | 3 |

**Patient No. 42**

Total no. of field Evaluated:5

| | | | | |
|---|---|---|---|---|
| Level of HIF 1-α | High: | | Low /UD: | |
| | 2 | | 3 | |
| Level of Oncostatin M | High: | Low/UD: | High: | Low/UD: |
| | 2 | 0 | 1 | 2 |

## Patient wise correlation between Oncostatin M Vs CD206

**Patient No. 3**

Total no. of field Evaluated: 11

| | | | | |
|---|---|---|---|---|
| Level of Oncostatin M | High: | | Low /UD: | |
| | 7 | | 4 | |
| Level of CD206 | High: | Low/UD: | High: | Low/UD: |
| | 7 | 0 | 0 | 4 |

**Patient No. 25**

Total no. of field Evaluated:3

| | | | | |
|---|---|---|---|---|
| Level of Oncostatin M | High: | | Low /UD: | |
| | 2 | | 1 | |
| Level of CD206 | High: | Low/UD: | High: | Low/UD: |
| | 2 | 0 | 0 | 1 |

**Patient No. 28**

Total no. of field Evaluated:5

| | | | | |
|---|---|---|---|---|
| Level of Oncostatin M | High: | | Low /UD: | |
| | 5 | | 0 | |
| Level of CD206 | High: | Low/UD: | High: | Low/UD: |
| | 5 | 0 | 0 | 0 |

**Patient No. 41**

Total no. of field Evaluated:5

| | | | | |
|---|---|---|---|---|
| Level of Oncostatin M | High: | | Low /UD: | |
| | 3 | | 2 | |
| Level of CD206 | High: | Low/UD: | High: | Low/UD: |
| | 3 | 0 | 0 | 2 |

**Patient No. 42**

Total no. of field Evaluated:5

| | | | | |
|---|---|---|---|---|
| Level of Oncostatin M | High: | | Low /UD: | |
| | 4 | | 1 | |
| Level of CD206 | High: | Low/UD: | High: | Low/UD: |
| | 4 | | 0 | 1 |

**Patient No.43**

Total no. of field Evaluated:5

| | High: | Low /UD: |
|---|---|---|
| Level of HIF 1-α | 2 | 3 |

| | High: | Low/UD: | High: | Low/UD: |
|---|---|---|---|---|
| Level of Oncostatin M | 2 | 0 | 0 | 3 |

**Patient No. 46**

Total no. of field Evaluated:10

| | High: | Low /UD: |
|---|---|---|
| Level of HIF 1-α | 7 | 3 |

| | High: | Low/UD: | High: | Low/UD: |
|---|---|---|---|---|
| Level of Oncostatin M | 7 | 0 | 0 | 3 |

**Patient No. 47**

Total no. of field Evaluated:2

| | High: | Low /UD: |
|---|---|---|
| Level of HIF 1-α | 1 | 1 |

| | High: | Low/UD: | High: | Low/UD: |
|---|---|---|---|---|
| Level of Oncostatin M | 1 | 0 | 0 | 1 |

**Patient No. 51**

Total no. of field Evaluated: 10

| | High: | Low /UD: |
|---|---|---|
| Level of HIF 1-α | 6 | 4 |

| | High: | Low/UD: | High: | Low/UD: |
|---|---|---|---|---|
| Level of Oncostatin M | 6 | 0 | 0 | 4 |

**Patient No. 52**

Total no. of field Evaluated:2

| | High: | Low /UD: |
|---|---|---|
| Level of HIF 1-α | 1 | 1 |

| | High: | Low/UD: | High: | Low/UD: |
|---|---|---|---|---|
| Level of Oncostatin M | 1 | 0 | 0 | 1 |

**Patient No. 56**

Total no. of field Evaluated:4

| | High: | Low /UD: |
|---|---|---|
| Level of HIF 1-α | 3 | 1 |

**Patient No. 43**

Total no. of field Evaluated:4

| | High: | Low /UD: |
|---|---|---|
| Level of Oncostatin M | 1 | 3 |

| | High: | Low/UD: | High: | Low/UD: |
|---|---|---|---|---|
| Level of CD206 | 1 | 0 | 0 | 3 |

**Patient No. 46**

Total no. of field Evaluated:3

| | High: | Low /UD: |
|---|---|---|
| Level of Oncostatin M | 1 | 2 |

| | High: | Low/UD: | High: | Low/UD: |
|---|---|---|---|---|
| Level of CD206 | 1 | 0 | 1 | 1 |

**Patient No. 47**

Total no. of field Evaluated:3

| | High: | Low /UD: |
|---|---|---|
| Level of Oncostatin M | 1 | 2 |

| | High: | Low/UD: | High: | Low/UD: |
|---|---|---|---|---|
| Level of CD206 | 1 | 0 | 0 | 2 |

**Patient No. 51**

Total no. of field Evaluated: 3

| | High: | Low /UD: |
|---|---|---|
| Level of Oncostatin M | 2 | 1 |

| | High: | Low/UD: | High: | Low/UD: |
|---|---|---|---|---|
| Level of CD206 | 2 | 0 | 0 | 1 |

**Patient No. 52**

Total no. of field Evaluated:2

| | High: | Low /UD: |
|---|---|---|
| Level of Oncostatin M | 1 | 1 |

| | High: | Low/UD: | High: | Low/UD: |
|---|---|---|---|---|
| Level of CD206 | 1 | 0 | 0 | 1 |

**Patient No. 56**

Total no. of field Evaluated:5

| | High: | Low /UD: |
|---|---|---|
| Level of Oncostatin M | 2 | 3 |

| | Level of | High: | Low/UD: | High: | Low/UD: |
|---|---|---|---|---|---|
| | Oncostatin M | 3 | 0 | 0 | 1 |

Total no. of field Evaluated:6

| Patient No. 57 | Level of | High: | Low /UD: | | |
|---|---|---|---|---|---|
| | HIF 1-α | 3 | 3 | | |
| | Level of | High: | Low/UD: | High: | Low/UD: |
| | Oncostatin M | 3 | 0 | 1 | 2 |

Total no. of field Evaluated:3

| Patient No. 59 | Level of | High: | Low /UD: | | |
|---|---|---|---|---|---|
| | HIF 1-α | 1 | 2 | | |
| | Level of | High: | Low/UD: | High: | Low/UD: |
| | Oncostatin M | 1 | 0 | 0 | 2 |

Total no. of field Evaluated:3

| Patient No. 60 | Level of | High: | Low /UD: | | |
|---|---|---|---|---|---|
| | HIF 1-α | 0 | 3 | | |
| | Level of | High: | Low/UD: | High: | Low/UD: |
| | Oncostatin M | 0 | 0 | 0 | 3 |

| | Level of | High: | Low/UD: | High: | Low/UD: |
|---|---|---|---|---|---|
| | CD206 | 2 | 0 | 0 | 3 |

Total no. of field Evaluated:6

| Patient No. 57 | Level of | High: | Low /UD: | | |
|---|---|---|---|---|---|
| | Oncostatin M | 2 | 4 | | |
| | Level of | High: | Low/UD: | High: | Low/UD: |
| | CD206 | 2 | 0 | 0 | 4 |

Total no. of field Evaluated:3

| Patient No. 59 | Level of | High: | Low /UD: | | |
|---|---|---|---|---|---|
| | Oncostatin M | 2 | 1 | | |
| | Level of | High: | Low/UD: | High: | Low/UD: |
| | CD206 | 2 | 0 | 0 | 1 |

Total no. of field Evaluated:2

| Patient No. 60 | Level of | High: | Low /UD: | | |
|---|---|---|---|---|---|
| | Oncostatin M | 0 | 2 | | |
| | Level of | High: | Low/UD: | High: | Low/UD: |
| | CD206 | 0 | 0 | 0 | 2 |

## Patient wise correlation between HIF-1α Vs Eotaxin

Total no. of field Evaluated: 6

| Patient No. 3 | Level of | High: | Low /UD: | | |
|---|---|---|---|---|---|
| | HIF 1-α | 4 | 2 | | |
| | Level of | High: | Low/UD: | High: | Low/UD: |
| | Eotaxin | 4 | 0 | 0 | 2 |

Total no. of field Evaluated:5

| Patient No. 25 | Level of | High: | Low /UD: |
|---|---|---|---|
| | HIF 1-α | 3 | 2 |

## Patient wise correlation between Eotaxin Vs CD206

Total no. of field Evaluated: 3

| Patient No. 3 | Level of | High: | Low /UD: | | |
|---|---|---|---|---|---|
| | Eotaxin | 2 | 1 | | |
| | Level of | High: | Low/UD: | High: | Low/UD: |
| | CD206 | 2 | 0 | 0 | 1 |

Total no. of field Evaluated:4

| Patient No. 25 | Level of | High: | Low /UD: |
|---|---|---|---|
| | Eotaxin | 2 | 2 |

| Patient | Level | High: | Low/UD: | High: | Low/UD: |
|---|---|---|---|---|---|
| | Level of Eotaxin | 3 | 0 | 0 | 2 |
| | Total no. of field Evaluated:5 | | | | |
| Patient No. 28 | Level of HIF 1-α | High: 2 | Low /UD: 3 | | |
| Patient No. 28 | Level of Eotaxin | 2 | 0 | 0 | 3 |
| | Total no. of field Evaluated:3 | | | | |
| Patient No. 41 | Level of HIF 1-α | High: 2 | Low /UD: 1 | | |
| Patient No. 41 | Level of Eotaxin | 2 | 0 | 0 | 1 |
| | Total no. of field Evaluated:6 | | | | |
| Patient No. 42 | Level of HIF 1-α | High: 3 | Low /UD: 3 | | |
| Patient No. 42 | Level of Eotaxin | 3 | 0 | 0 | 3 |
| | Total no. of field Evaluated:6 | | | | |
| Patient No.43 | Level of HIF 1-α | High: 3 | Low /UD: 3 | | |
| Patient No.43 | Level of Eotaxin | 3 | 0 | 1 | 2 |
| | Total no. of field Evaluated:4 | | | | |
| Patient No. 46 | Level of HIF 1-α | High: 4 | Low /UD: 0 | | |
| Patient No. 46 | Level of Eotaxin | 4 | 0 | 0 | 0 |
| Pa tie . | Total no. of field Evaluated:4 | | | | |

| Patient | Level | High: | Low/UD: | High: | Low/UD: |
|---|---|---|---|---|---|
| | Level of CD206 | 2 | 0 | 0 | 2 |
| | Total no. of field Evaluated:5 | | | | |
| Patient No. 28 | Level of Eotaxin | High: 3 | Low /UD: 2 | | |
| Patient No. 28 | Level of CD206 | 3 | 0 | 0 | 2 |
| | Total no. of field Evaluated:4 | | | | |
| Patient No. 41 | Level of Eotaxin | High: 2 | Low /UD: 2 | | |
| Patient No. 41 | Level of CD206 | 2 | 0 | 0 | 2 |
| | Total no. of field Evaluated:3 | | | | |
| Patient No. 42 | Level of Eotaxin | High: 2 | Low /UD: 1 | | |
| Patient No. 42 | Level of CD206 | 1 | 1 | 0 | 1 |
| | Total no. of field Evaluated:4 | | | | |
| Patient No. 43 | Level of Eotaxin | High: 2 | Low /UD: 2 | | |
| Patient No. 43 | Level of CD206 | 2 | 0 | 0 | 2 |
| | Total no. of field Evaluated:3 | | | | |
| Patient No. 46 | Level of Eotaxin | High: 2 | Low /UD: 1 | | |
| Patient No. 46 | Level of CD206 | 2 | 0 | 1 | 0 |
| Pa tie | Total no. of field Evaluated:3 | | | | |

| Level of HIF 1-α | High: 2 | | Low /UD: 2 | |
|---|---|---|---|---|
| Level of Eotaxin | High: 2 | Low/UD: 0 | High: 1 | Low/UD: 1 |

Total no. of field Evaluated: 5

| Level of Eotaxin | High: 2 | | Low /UD: 1 | |
|---|---|---|---|---|
| Level of CD206 | High: 2 | Low/UD: 0 | High: 0 | Low/UD: 1 |

Total no. of field Evaluated: 5

**Patient No. 51**

| Level of HIF 1-α | High: 3 | | Low /UD: 2 | |
|---|---|---|---|---|
| Level of Eotaxin | High: 3 | Low/UD: 0 | High: 0 | Low/UD: 2 |

Total no. of field Evaluated:4

**Patient No. 51**

| Level of Eotaxin | High: 3 | | Low /UD: 2 | |
|---|---|---|---|---|
| Level of CD206 | High: 3 | Low/UD: 0 | High: 0 | Low/UD: 2 |

Total no. of field Evaluated:5

**Patient No. 52**

| Level of HIF 1-α | High: 1 | | Low /UD: 3 | |
|---|---|---|---|---|
| Level of Eotaxin | High: 1 | Low/UD: 0 | High: 0 | Low/UD: 3 |

Total no. of field Evaluated:4

**Patient No. 52**

| Level of Eotaxin | High: 2 | | Low /UD: 3 | |
|---|---|---|---|---|
| Level of CD206 | High: 2 | Low/UD: 0 | High: 0 | Low/UD: 3 |

Total no. of field Evaluated:3

**Patient No. 56**

| Level of HIF 1-α | High: 3 | | Low /UD: 1 | |
|---|---|---|---|---|
| Level of Eotaxin | High: 3 | Low/UD: 0 | High: 0 | Low/UD: 1 |

Total no. of field Evaluated:5

**Patient No. 56**

| Level of Eotaxin | High: 2 | | Low /UD: 1 | |
|---|---|---|---|---|
| Level of CD206 | High: 2 | Low/UD: 0 | High: 0 | Low/UD: 1 |

Total no. of field Evaluated:4

**Patient No. 57**

| Level of HIF 1-α | High: 2 | | Low /UD: 3 | |
|---|---|---|---|---|
| Level of Eotaxin | High: 2 | Low/UD: 0 | High: 0 | Low/UD: 3 |

Total no. of field Evaluated:5

**Patient No. 57**

| Level of Eotaxin | High: 3 | | Low /UD: 1 | |
|---|---|---|---|---|
| Level of CD206 | High: 2 | Low/UD: 1 | High: 0 | Low/UD: 1 |

Total no. of field Evaluated:4

**Patient No. 59**

| Level of HIF 1-α | High: 2 | | Low /UD: 3 | |
|---|---|---|---|---|
| Level of Eotaxin | High: | Low/UD: | High: | Low/UD: |

**Patient No. 59**

| Level of Eotaxin | High: 2 | | Low /UD: 2 | |
|---|---|---|---|---|
| Level of CD206 | High: | Low/UD: | High: | Low/UD: |

| Patient No. 60 | | | | | |
|---|---|---|---|---|---|
| | | 2 | 0 | 0 | 3 |
| | | Total no. of field Evaluated:3 | | | |
| | Level of HIF 1-α | High: 2 | | Low /UD: 1 | |
| | Level of Eotaxin | High: 2 | Low/UD: 0 | High: 0 | Low/UD: 1 |

| Patient No. 60 | | | | | |
|---|---|---|---|---|---|
| | | 2 | 0 | 0 | 2 |
| | | Total no. of field Evaluated:4 | | | |
| | Level of Eotaxin | High: 2 | | Low /UD: 2 | |
| | Level of CD206 | High: 2 | Low/UD: 0 | High: 0 | Low/UD: 2 |

**Numbers represent fields showing presence of indicated antigen evaluated; UD =Undetectable level.**

## Suppl. 5: Summary of Immunohistochemical Observations of Individual Human Breast Cancer Specimen

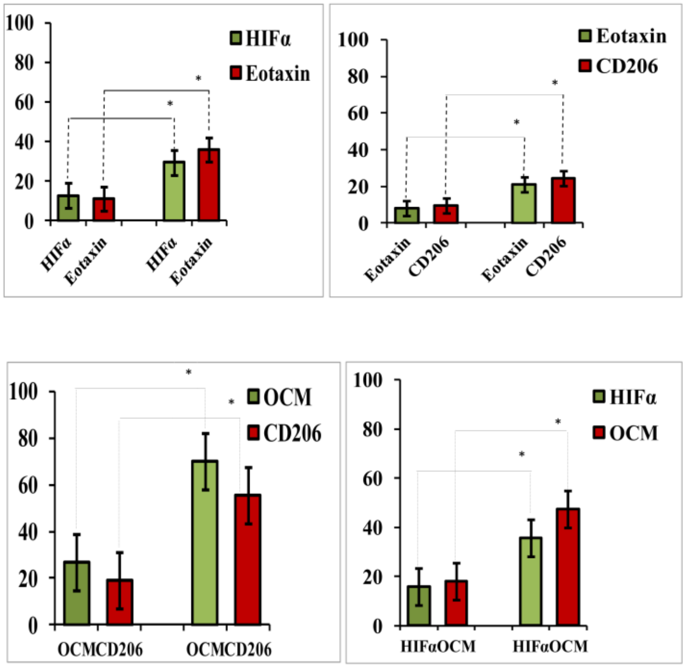


Each bar represents the mean intensity of five different fields.

**Suppl.6: Quantification of Oncostatin M/Eotaxin and CD206 Expression (Florescence intensity) in Hypoxic (HIF-1α Positive) area of Human Breast Cancer Specimen**

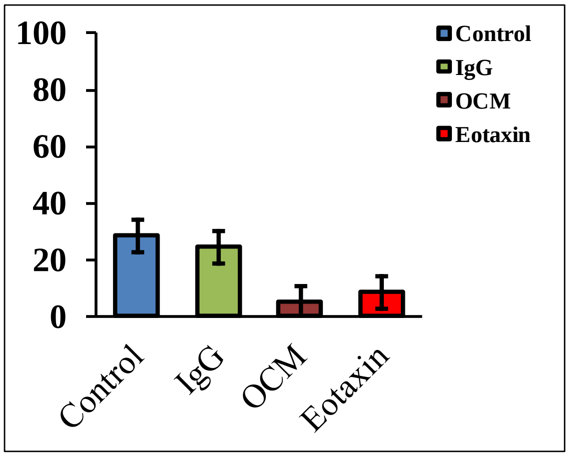


Each bar represents the mean intensity of five different fields.

**Suppl.7: Quantification of CD31Expression (Florescence intensity) in 4T1/BALB/c Tumour Tissue**

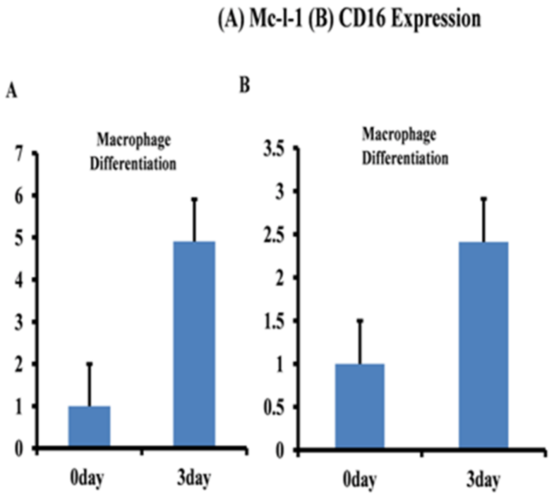


**Suppl. 8: Real-Time Quantitative PCR of Macrophage Specific Markers in THP-1 Derived Macrophages**
